# Supplementary material for: Atelocollagen exhibits superior performance compared to growth factors in upregulating proteins associated with tendon healing
Source: PLoS One. 2025 Sep 10;20(9):e0323156. doi: 10.1371/journal.pone.0323156 (PMC12422506; doi:10.1371/journal.pone.0323156)
Supplement: Attachment — Submitted filename: report_10.1101+2025.04.06.647440.pdf [file pone.0323156.s002.pdf]

ScreenIT Report

This is an automated report for 10.1101+2025.04.06.647440.pdf. This report was solicited by the PLOS One editorial team and provided by ScreenIT.

ScreenIT is an independent group of scientists developing automated tools that analyze academic papers. A set of automated tools screened your submitted manuscript and provided the report below. Each tool was created by your academic colleagues with the goal of helping authors.

The tools look for factors that are important for transparency, rigor and reproducibility, and we hope that the report might help you to improve reporting in your manuscript. Within the report you will find links to more information about the items that the tools check. These links include helpful papers, websites, or videos that explain why the item is important.

While our screening tools aim to improve and maintain quality standards they may, on occasion, miss nuances specific to your study type or flag something incorrectly. Each tool has limitations that are described on the ScreenIT website. The tools screen the main file for the paper; they are not able to screen supplements stored in separate files.

Please note that the Academic Editor had access to these comments while making a decision on your manuscript. The Academic Editor may ask that issues flagged in this report be addressed.

If you would like to provide feedback on the ScreenIT tool, please email the team at ScreenIt@bih-charite.de. If you have questions or concerns about the review process, please contact the PLOS One office at plosone@plos.org.

How to read the report:

- ✓ - no action required
- ? - review for potential issues
- ! - a critical issue was found by a tool
- n/a - may not be applicable to your paper

- more information about this item can be obtained using this link

Rigor

n/a	Ethics	not required.
n/a	Inclusion and Exclusion Criteria	not required.
?	Flow charts and attrition	We did not find a study flow chart of excluded observations. We strongly recommend using flow charts because they provide an overview of the study design and more information about attrition. If you included a study flow chart in your supplemental files, we apologize for missing this. Our tool is not able to screen separate supplemental files.<br><br>Sentence about attrition: *"not required."*
?	Sex as a biological variable	Not detected. Please consider reporting the sex of human participants, animals or cells, if you haven't done so.
✓	Subject Demographics	Weight: *"Equal amounts of protein (2030 g) were loaded onto SDS-PAGE gels (1012%) and separated by electrophoresis at 100V for 90 minutes."*
?	Randomization	Not detected. If you performed an experimental study, please specify whether participants, animals or samples were randomly assigned to treatments or groups, and specify the randomization procedure.
?	Blinding	Not detected. Please specify whether blinding was used at various phases of the experiment (e.g., blinding of patients, caregivers, outcome assessments, data analysis).
?	Power Analysis	Not detected. Please state how you determined the sample size for the study. If you performed a power or sample size calculation, please provide details.
✓	Replication	*"All experiments were independently repeated three times."*<br>Number: *"All experiments were independently repeated three times."*

?	Cell Line Authentication	not detected.

Generated by [*Barzooka (flow charts)*](), [*SciScore (everything else)*]().
SciScore only screens the text of the methods section; we apologize if the tool has missed items reported elsewhere in the paper or supplements.

Resources

Persistent unique identifiers for research resources, RRIDs, can be used to track these key resources though the literature. They are requested or required in many journals, but even if your journal does not require them, we strongly encourage the use of these for the readers of papers who wish to easily track down the key materials like antibodies, software tools, or mice.

Antibodies	*"Membranes were incubated overnight at 4C with primary antibodies against p-ERK (Thr202/Tyr204; rabbit monoclonal, Cell Signaling Technology, Cat. No. 9101, Danvers, MA, USA), ERK1/2 (rabbit monoclonal, Cell Signaling Technology, Cat. No. 9102)"*	? p-ERK suggested: None. Please add an RRID to help others identify this resource. ? ERK1/2 suggested: None. Please add an RRID to help others identify this resource.
Antibodies	*"Primary antibodies targeting -actin (cytoskeletal marker, red fluorescence) and DAPI (nuclear stain, blue fluorescence) were applied overnight at 4C in 1% BSA in PBS."*	? -actin suggested: None. Please add an RRID to help others identify this resource.
Antibodies	*"Cells were washed three times with PBS and incubated with fluorescently labeled secondary antibodies (Alexa Fluor 594 for actin and DAPI for nuclei) for 1 hour at room temperature in the dark."*	? fluorescently labeled secondary antibodies (Alexa Fluor 594 for actin suggested: None. Please add an RRID to help others identify this resource.

Experimental Models: Cell Lines	*"Western blot analysis Western blot analysis was performed to evaluate the expression of key signaling, muscle differentiation, ECM, tendon, adipogenic, and mitochondrial proteins.C2C12 cells were cultured and treated with Atelocollagen (AC, 1nM and 10nM) or individual growth factors (BMP-2, TGF-1, IGF1, VEGF, each at 1nM and 10nM) for 6 days [15-18]."*	proteins.C2C12 suggested: None. Please add an RRID to help others identify this resource.
Experimental Models: Cell Lines	*"Immunofluorescence This experiment aimed to evaluate cytoskeletal organization and actin filament distribution in C2C12 cells following treatment with AC and growth factors (VEGF and TGF-1) using immunofluorescence staining."*	C2C12 suggested: RRID:CVCL_0188. Please make sure this RRID suggestion is accurate before including in your manuscript.
Software and Algorithms	*"Fluorescence intensity was quantified using ImageJ software, with actin expression normalized to nuclear staining."*	ImageJ suggested: ImageJ, RRID:SCR_003070. Please make sure this RRID suggestion is accurate before including in your manuscript.
Software and Algorithms	*"Statical analysis All statistical analyses were performed using SPSS software (IBM SPSS Statistics, version XX, Armonk, NY, USA)."*	SPSS suggested: SPSS, RRID:SCR_002865. Please make sure this RRID suggestion is accurate before including in your manuscript.

Generated by SciScore.
SciScore only screens the text of the methods section; we apologize if the tool has missed items reported elsewhere in the paper or supplements.

Transparency

?	Open code	If you wrote code to analyze your data, please consider sharing the code in a public repository. This makes it easier for others to reproduce your analyses, and may also aid others seeking to analyze similar datasets.
?	Open data	If permitted, sharing data on a public repository with a persistent identifier (a DOI or accession number) can improve reproducibility and make it easier for other scientists to expand on your work. Papers with open data are cited more often than papers without open data. Some institutions have an expert who can provide advice on data sharing.
✓	Self-acknowledged limitations	*"Despite these promising results, this study has several limitations. First, the experiments were conducted in an in vitro model, which does not fully replicate the complex biomechanical and biochemical environment of in vivo tendon healing. Second, only a single concentration (10nM) was evaluated in depth, whereas a dose-dependent study in an in vivo setting may provide additional insights into ACs therapeutic potential. Furthermore, long-term studies assessing mechanical strength and functional outcomes in tendon repair models are necessary to confirm the translational applicability of AC in clinical settings."*
?	Conflict of interest statement	It is important to supply a conflict of interest statement, even if there are none to declare, to transparently present the research.
✓	Funding statement	Thank you for reporting your funding source(s), or stating that there was no specific funding for this work.
n/a	Registration statement	A registered protocol makes the planned research project more transparent and can protect against bias. Please consider registering future confirmatory studies.

Generated by ODDPub, limitation-recognizer, rtransparent, trial-identifier.

Figures

	Bar graphs of continuous data	We found bar graphs of continuous data. We recommend replacing these with more informative graphics (e.g. dotplots, box plots, violin plots), as many different datasets can lead to the same bar graph. The actual data may suggest different conclusions from the summary statistics alone. Please see the following resources for more information on the problems with bar graphs (Weissgerber et al 2015) and what to use instead (Weissgerber et al 2019).
	No rainbow color maps	Thank you for not using rainbow colormaps. Rainbow color maps distort readers' perception of the data by introducing visual artifacts and are not accessible to colorblind readers.

Generated by JetFighter, Barzooka.
