# Supplementary material for: Atelocollagen exhibits superior performance compared to growth factors in upregulating proteins associated with tendon healing
Source: PLoS One. 2025 Sep 10;20(9):e0323156. doi: 10.1371/journal.pone.0323156 (PMC12422506; doi:10.1371/journal.pone.0323156)
Supplement: Attachment — Submitted filename: Response to Reviewers.docx [file pone.0323156.s004.docx]

5. Review Comments to the Author

Please use the space provided to explain your answers to the questions above. You may also include additional comments for the author, including concerns about dual publication, research ethics, or publication ethics. (Please upload your review as an attachment if it exceeds 20,000 characters)

Reviewer #1: Manuscript ID: PONE-D-25-17647
Title: Atelocollagen exhibits superior performance compared to growth factors in upregulating proteins associated with tendon healing

This manuscript presents an in vitro study evaluating the efficacy of atelocollagen (AC) compared to several individual growth factors (BMP-2, TGF-β1, IGF-1, and VEGF) on molecular markers associated with tendon healing. The experimental design, including Western blotting, MTT assay, and immunofluorescence, is generally robust. The manuscript is well-written and contributes novel insights into the potential of AC as a biological augmentation strategy. However, there are minor areas that require clarification and revision before acceptance.


1. Introduction

The introduction clearly outlines the clinical need for enhanced tendon-to-bone interface healing and the limitations of current biological augmentation strategies.
The rationale for using growth factors such as BMP-2, TGF-β1, IGF-1, and VEGF is well described, and the transition to discussing Atelocollagen as an alternative is logical.

However, to better emphasize the novelty of this study, the authors should more clearly highlight the limitations of previous literature. In particular, the authors are encouraged to underscore that, to date, no published studies have directly examined or compared the effects of atelocollagen on molecular markers relevant to tendon healing—especially in the context of rotator cuff tears—making the current investigation uniquely valuable.
Response: We thank the reviewer for this valuable comment. In response, we have revised the Introduction section to more clearly highlight the limitations of previous growth factor-based therapies, including their short half-life, rapid degradation, and challenges in controlled delivery. We have also underscored that, to date, no published studies have directly examined or compared the effects of atelocollagen on tendon healing-related molecular markers, particularly in the context of rotator cuff tears. These revisions aim to better highlight the novelty and significance of our work. The updated Introduction is included below for your review.

Lines 66-74: “Among these, therapies based on exogenous growth factors such as bone morphogenetic protein-2 (BMP-2), transforming growth factor-beta 1 (TGF-β1), insulin-like growth factor-1 (IGF-1), and vascular endothelial growth factor (VEGF) have shown the potential to stimulate ECM remodeling, promote tendon cell proliferation, and enhance collagen synthesis [7-9] However, previous literature has highlighted critical limitations of these growth factor therapies, including their rapid degradation, short biological half-life, and difficulties in achieving sustained and localized delivery [8, 10, 11]. Consequently, despite promising experimental data, their clinical translation has been limited by the inability to maintain therapeutic concentrations at the repair site over time.” was added.

Lines 75-83: The original sentences were deleted.

Lines 84-93: “Given these challenges, alternative strategies that provide more sustained and controlled biological augmentation are urgently needed. Atelocollagen (AC), a highly purified collagen derivative, has emerged as a promising biomaterial for tendon repair due to its structural stability, biocompatibility, and inherent bioactivity [12]. Unlike exogenous growth factors, AC possesses a sustained bioactive profile and does not require additional carrier systems to maintain its therapeutic effects. Despite its promising biological properties, there is a paucity of data comparing the efficacy of AC with that of individual growth factors in regulating molecular markers critical for tendon healing [13]. In particular, no studies to date have directly examined or compared the effects of AC on key tendon healing-related molecular markers such as phosphorylated extracellular signal-regulated kinase (p-ERK), collagen type I (Col I), collagen type III (Col III), and tenascin C (TnC), especially within the context of rotator cuff tears.” was added.

Lines 94-101: The original sentences were deleted.

The hypothesis is clearly stated
Response: We appreciate the reviewer’s comment. To ensure that the hypothesis is explicitly and clearly stated in the manuscript, we have refined the wording in the Introduction section. This revision ensures that the hypothesis is both clear and concise for the reader.

Lines 103-106: The original sentences were deleted.

Lines 106-109: “We hypothesize that AC would have comparable or superior effects to individual growth factors in upregulating p-ERK, Col I, Col III, and TnC, thus highlighting its potential as a robust biological augmentation strategy for TBI healing following surgical repair.” was added.

2. Materials and Methods
Lines 90-91: Please justify the use of C2C12 cells instead of tenocytes or tendon-derived stem cells. This point should also be addressed in the discussion.
Response: Thank you for your insightful comment. We acknowledge the concern regarding the choice of C2C12 myoblasts instead of tenocytes or tendon-derived stem cells (TDSCs). C2C12 cells, while not primary tenocytes, are a well-established myogenic cell line with robust proliferative and differentiation capacity. They exhibit myogenic-to-tenogenic trans differentiation potential when subjected to mechanical or biochemical stimuli, and are frequently used as a surrogate model in tendon research due to their consistent growth characteristics and reproducible responses.^a,b)^ Furthermore, their use facilitates the standardization and reproducibility of experiments, which is crucial for elucidating the comparative molecular effects of AC and growth factors in an in vitro environment. We have added this justification to both the Materials and Methods and the Discussion sections (limitation section).

a) Lautaoja JH, Pekkala S, Pasternack A, Laitinen M, Ritvos O, Hulmi JJ. Differentiation of Murine C2C12 Myoblasts Strongly Reduces the Effects of Myostatin on Intracellular Signaling. Biomolecules. 2020;10(5):695.

b) Uemura K, Hayashi M, Itsubo T, Oishi A, Iwakawa H, Komatsu M, Uchiyama S, Kato H. Myostatin promotes tenogenic differentiation of C2C12 myoblast cells through Smad3. FEBS Open Bio. 2017 Feb 20;7(4):522-532.

Lines 117-119: “Although they are not primary tendon cells, they have been extensively validated as a surrogate model for tenogenic signaling and differentiation under tendon-specific stimuli, ensuring high reproducibility, standardized cell expansion, and reliable molecular response quantification.” was added.

Lines 344-347: “First, although C2C12 myoblasts are a well-established and reproducible in vitro model for investigating tendon-specific signaling and tenogenic differentiation under biochemical or mechanical stimuli, they do not fully recapitulate the cellular and extracellular matrix interactions of primary tenocytes or tendon-derived stem cells.”

Line 145–150: There is a typographical error in the section header
"Statical analysis" → should be corrected to "Statistical analysis"
Response: We have corrected the typographical error from "Statical analysis" to "Statistical analysis."

3. Results
Lines 170–176:
Only IGF-1 group showed a statistically lower ratio compared to AC on col I/III ratio.
Consider discussing the biological relevance of this change more in the Results or Discussion.
Response: Thank you for your valuable comment. We appreciate the opportunity to clarify the biological relevance of the finding that only the IGF group showed a statistically lower Col I/III ratio compared to the AC group. This result suggests that while IGF may support some aspects of tendon healing, it does not effectively promote the ECM reorganization toward a more biomechanically robust state, as indicated by the lower Col I/III ratio. In contrast, the significant enhancement of this ratio in the AC-treated group underscores AC’s superior ability to foster a more mature and load-bearing collagen matrix. This difference is crucial because a higher Col I/III ratio is associated with improved tendon mechanical strength and long-term functional recovery. We have integrated this interpretation into the revised Discussion section to highlight the unique advantage of AC over IGF in this context.

Lines 280-285: The original sentences were deleted.

Lines 286-297: “Notably, our results demonstrated that only the IGF group showed a statistically lower Col I/III ratio compared to the AC group, while no significant differences were found between AC and the other growth factors. This selective enhancement suggests that AC may overcome IGF’s limitations in promoting biomechanical matrix integrity. A lower Col I/III ratio, as seen with IGF treatment, is associated with less organized and weaker ECM, potentially compromising tendon mechanical strength. In contrast, the significant increase in the Col I/III ratio in the AC-treated group indicates a shift toward a more mature, load-bearing collagen composition that supports improved tendon function. These findings collectively underscore that AC not only promotes ECM remodeling but also uniquely enhances the formation of a biomechanically robust tendon matrix compared to IGF alone. Given that clinical studies have linked a higher Col I/III ratio to improved mechanical properties and reduced retear rates, this highlights AC’s potential as an effective biological augmentation strategy for tendon healing.” was added.

4. Discussion
It would also strengthen the manuscript if the authors could cite additional references supporting the beneficial effects of atelocollagen in rotator cuff repair, to provide a broader context for the clinical relevance of their findings. Moreover, discussing the limitations of those previous studies would help clarify how the present study addresses existing gaps in the literature.
Response: Thank you for your valuable comment. In response, we have added additional references supporting the beneficial effects of atelocollagen in rotator cuff repair to provide a broader clinical context. Furthermore, we have included a brief discussion of the limitations of these previous studies to highlight how the present study addresses existing gaps in the literature. These additions strengthen the overall clinical relevance of our findings and underscore the translational potential of atelocollagen as a biological augmentation strategy.

Lines 326-336: “Atelocollagen has garnered considerable attention as a promising biomaterial in the context of rotator cuff repair, with preclinical evidence suggesting its ability to enhance TBI healing and improve biomechanical properties [12, 13]. Importantly, Suh et al., reported that atelocollagen scaffolds significantly augmented structural stability and biocompatibility in TBI healing models [37]. Nevertheless, these investigations have largely been confined to short-term evaluations and have not directly compared AC with alternative biological augmentation strategies, thereby limiting the broader applicability of their findings. In contrast, the present study seeks to address these limitations by examining key molecular markers of tendon healing while directly comparing AC to growth factor-based therapies. This approach offers valuable insights into the capacity of AC to foster tendon-specific cellular responses and orchestrate matrix remodeling, highlighting its potential as a refined strategy for biological augmentation in tendon repair.” was added.

Line 274: Please remove or revise Line 274, unless supporting angiogenesis data are provided.
Response: Thank you for your helpful comment. In response, we have removed the mention of neovascularization (angiogenesis) from the discussion of atelocollagen’s effects to focus exclusively on the primary molecular mechanisms examined in this study, including ERK activation, TnC upregulation, and improved Col I/III ratio.

Line 338: “VEGF-mediated neovascularization” was deleted.

5. Conclusion
The conclusion is consistent with the results and maintains a modest tone.
Response: Thank you for your positive feedback and for acknowledging the clarity and modest tone of our conclusions.

6. Figures and Table
Please spell out all abbreviations in the figure legends or provide explanatory footnotes to ensure clarity for the reader.
Response: Thank you for your suggestion. We have revised the figure legends and table footnotes to spell out all abbreviations, ensuring that readers can easily understand the figures and data presented.

Lines 638-639: “p-ERK, phosphorylated extracellular signal-regulated kinase; AC, atelocollagen; BMP-2, bone morphogenetic protein-2; IGF-1, insulin-like growth factor, SD; standard deviation.” was added.

Lines 643-646: “AC, atelocollagen; BMP-2, bone morphogenetic protein-2; IGF-1, insulin-like growth factor-1; VEGF, vascular endothelial growth factor; TGF-β1, transforming growth factor-beta 1; TnC, tenascin C; SD, standard deviation.” was added.

Lines 650-651: “AC, atelocollagen; IGF-1, insulin-like growth factor-1; Col I/III, collagen type I/III; ECM, extracellular matrix; SD, standard deviation” was added.

Lines 656-657: “AC, atelocollagen; VEGF, vascular endothelial growth factor; TGF-β1, transforming growth factor-beta 1” was added.

Lines 664-666: “AC, atelocollagen; VEGF, vascular endothelial growth factor; TGF-β1, transforming growth factor-beta 1; F-actin, filamentous actin.” was added.

Lines 668-669: “. AC, atelocollagen; VEGF, vascular endothelial growth factor; TGF-β1, transforming growth factor-beta 1; SD, standard deviation.”

7. References
Reference formatting and selection are appropriate.

Response: Thank you for your positive feedback and for acknowledging the appropriateness of the reference formatting and selection.

Reviewer #2: This is an automated report for PONE-D-25-17647. This report was solicited by the PLOS One editorial team and provided by ScreenIT.
ScreenIT is an independent group of scientists developing automated tools that analyze academic papers. A set of automated tools screened your submitted manuscript and provided the report below. Each tool was created by your academic colleagues with the goal of helping authors. The tools look for factors that are important for transparency, rigor and reproducibility, and we hope that the report might help you to improve reporting in your manuscript. Within the report you will find links to more information about the items that the tools check. These links include helpful papers, websites, or videos that explain why the item is important. While our screening tools aim to improve and maintain quality standards they may, on occasion, miss nuances specific to your study type or flag something incorrectly. Each tool has limitations that are described on the ScreenIT website. The tools screen the main file for the paper; they are not able to screen supplements stored in separate files. Please note that the Academic Editor had access to these comments while making a decision on your manuscript. The Academic Editor may ask that issues flagged in this report be addressed. If you would like to provide feedback on the ScreenIT tool, please email the team at [ScreenIt@bih-charite.de](mailto:ScreenIt@bih-charite.de). If you have questions or concerns about the review process, please contact the PLOS One office at [plosone@plos.org](mailto:plosone@plos.org).

Reviewer #3: As the development is a gradual and progressive process, assessment of tendon development in one point (only day 5) is not reasonable and sufficient.
So, it is strongly recommend to perform the analysis and asses the gene expression in different time points (such as 3, 7, 14 and 21 days after culture) and longer time for the special genes (genes express in earlier or final development stage) in each phase.

Response: Thank you for your constructive feedback regarding the temporal aspects of tendon development assessment. We agree that tendon healing and regeneration involve a dynamic and time-dependent process, and we recognize the value of analyzing gene expression at multiple time points to capture the temporal progression of molecular events. However, in the present study, we aimed to establish an initial comparative analysis of AC versus individual growth factors on key tendon-healing markers at a standardized time point (day 5) based on prior work demonstrating maximal marker expression at this time. We acknowledge that a time-course analysis at additional time points (such as 3, 7, 14, and 21 days) would provide a more comprehensive understanding of the molecular dynamics involved in tendon healing and could be an important direction for future studies. We have included this point in the revised Discussion section to highlight this limitation and to suggest it as a potential avenue for further research.

Lines 351-353: The original sentences were deleted.

Lines 353-359: “Furthermore, our analysis was limited to a single time point (day 5), which does not fully capture the dynamic, time-dependent nature of tendon healing. Future studies assessing gene expression and molecular marker regulation at multiple time points (such as 3, 7, 14, and 21 days) would provide a more comprehensive understanding of the molecular and functional changes over time. Finally, long-term studies assessing mechanical strength and functional outcomes in tendon repair models are necessary to confirm the translational applicability of AC in clinical settings.” was added.

**ScreenIT**

Response: Thank you for your suggestion regarding the inclusion of a study flow chart. In response, we have added a detailed flowchart to the revised manuscript to provide a clear visual overview of the experimental design, including the treatment assignments and subsequent analytical processes.

Regarding attrition, there were no excluded samples or dropped observations during the experiment. All assigned samples (n = 9 in total) were successfully allocated to the three assay groups (Western blot, MTT, and immunofluorescence) and analyzed as planned. Therefore, no attrition-specific notation was required in this case.

Fig 1.

Lines 611-620: “Figure 1. Experimental workflow for C2C12 myoblast analysis. This flowchart summarizes the experimental design used to evaluate the effects of AC and growth factors on C2C12 myoblasts. The cultured cells were divided into three experimental groups (n = 3) and subjected to MTT assay (for cell proliferation), Western blot analysis (for p-ERK/ ERK, Col I/III, and TnC), and immunofluorescence staining (for actin and nuclear staining). All experiments were independently repeated three times. Data were quantified using ImageJ software, and statistical analysis was performed using one-way ANOVA followed by Tukey’s post hoc test in SPSS. A p-value of less than 0.05 was considered statistically significant.” was added.

Response: Thank you for pointing this out. We would like to clarify that the cell line used in this study, C2C12, is a murine (mouse) myoblast cell line derived from male C3H mice, and this is now explicitly stated in the Methods section of the revised manuscript.

Lines 114-115: “C2C12 cells, a male murine myoblast cell line derived from C3H mice (KCTC, Cat. No. KCTC-10410), were used in this study.” was added.

Response: Thank you for the valuable comment. Although this study involved an in vitro experiment using the C2C12 myoblast cell line, we took steps to minimize bias. After culturing, cells were randomly assigned to treatment groups using a blinded labeling system, in which treatment solutions were coded and applied without knowledge of group identity. This ensured objectivity in the treatment administration process. This information has been added to the Methods section of the revised manuscript.

Lines 131-134: “To minimize potential bias, cells were randomly allocated into experimental groups after expansion using a blinded labeling system. Treatment solutions (atelocollagen and growth factors) were prepared and coded by an independent researcher, and applied without the experimenter knowing the group assignments.” was added.

Response: We appreciate the reviewer’s insightful comment. In response, we calculated the required sample size based on effect sizes reported in previous studies with similar experimental conditions. These studies demonstrated adequate statistical power (≥ 0.80) with a sample size of *n = 3 per group* when using molecular assays such as Western blot, MTT proliferation analysis, and immunofluorescence imaging. Accordingly, we confirmed that a sample size of n = 3 per group was sufficient to detect statistically meaningful differences in our study design. All experiments were independently repeated three times to ensure reproducibility and robustness.

Lines 179-183: “Sample size justification was based on previous studies reporting detectable effect sizes using similar cell-based molecular assays (e.g., cell proliferation, western blotting, and Immunofluorescence). A minimum of three independent biological replicates (n = 3) per group was determined to be sufficient to detect statistically significant differences with a power of 0.80 and α = 0.05. All experiments were independently repeated three times.” was added.

Response: We thank the reviewer for pointing this out. The C2C12 murine myoblast cell line used in this study was obtained from the Korean Collection for Type Cultures (KCTC, Cat. No. KCTC-10410). The cell line is well-characterized, widely validated in the literature, and was authenticated by the supplier. No cross-contamination or misidentification has been reported for this batch. We have added this information to the methods section.

Lines 114-115: “C2C12 muscle cells were obtained from Korean Collection for Type Cultures (KCTC, Cat. No. KCTC-10410, Jeongeup, Korea). The C2C12 cells (RRID:CVCL_0188) is authenticated and confirmed to be free from mycoplasma contamination, according to the provider’s quality control certification.” was added.

Response: We thank the reviewer for highlighting the need for antibody resource identification. In the revised manuscript, we have added RRIDs for all primary and secondary antibodies used in Western blot and immunofluorescence experiments.

Lines 151-153,170-173:

p-ERK (Cell Signaling Technology, Cat. No. 9101, RRID:AB_331646)

ERK1/2 (Cell Signaling Technology, Cat. No. 9102, RRID:AB_330744)

β-actin (Sigma-Aldrich, Cat. No. A5441, RRID:AB_476744)

DAPI (Thermo Fisher, Cat. No. D1306, RRID:AB_2629482)

Alexa Fluor 594 goat anti-rabbit IgG (Thermo Fisher, Cat. No. A-11012, RRID:AB_2534079)

Response: Thank you for your comment. The C2C12 cell line used in this study is a murine myoblast cell line originally derived from a male C3H mouse. In the revised manuscript, we have now included the correct RRID (RRID:CVCL_0188).

Lines 114: “The C2C12 cells (RRID:CVCL_0188) is authenticated…..” was added.

Response: Thank you for your comment. We have now added the appropriate RRIDs for all software tools used in this study to enhance reproducibility. ImageJ (RRID:SCR_003070) was used for image quantification, and SPSS (RRID:SCR_002865) was used for statistical analysis.

Lines 159-160: “Densitometry analysis was performed using ImageJ software (version 1.53, National Institutes of Health, Bethesda, MD, USA; RRID:SCR_003070),” was added.

Lines 184-185: “SPSS software (IBM SPSS Statistics, version XX27, Armonk, NY, USA; RRID:SCR_002865).” was added.

Response: No custom code was used for data analysis in this study, and all data generated are included in the manuscript and its supplementary files.

Response: We appreciate the reminder. In accordance with the journal’s guidelines, a conflict of interest statement has been included in the revised manuscript and also explicitly stated in the cover letter submitted alongside the revised version. The authors declare that there are no conflicts of interest related to this work.

Response: Thank you for your thoughtful recommendation. We understand the concern regarding the use of bar graphs for continuous data. However, in this study, all measurements were taken at a single time point and represent a single averaged value per sample group. Therefore, the use of bar graphs with mean ± standard deviation (SD) most appropriately reflects the structure of the dataset and the experimental design.
